# Macrophage activation syndrome in a patient with juvenile systemic lupus erythematosus

**DOI:** 10.1186/1546-0096-12-S1-P221

**Published:** 2014-09-17

**Authors:** Violetta Opoka-Winiarska, Sara Piłat, Beata Polkowska

**Affiliations:** 1 Department of Pediatric Pulmonology and Rheumatology, Medical University of Lublin, Lublin, Poland; 2 Student Scientific Circles in Department of Pediatric Pulmonology and Rheumatology, Medical University of Lublin, Lublin, Poland

## Introduction

Macrophage activation syndrome (MAS) is a rare but life threatening complication of infectious, neoplastic and rheumatic diseases. Clinically, patients usually have high fever, hepatosplenomegaly, lymphadenopathy and neurologic symptoms. In laboratory results pathognomonic are pancytopenia, decline in the value of sedimentation rate (ESR), increased levels of ferritin and coagulation abnormalities.

## Objectives

The aim of the study is to present the case of Macrophage Activation Syndrome in a Patient With Juvenile Systemic Lupus Erythematosus.

## Methods

The clinical course and results of the diagnostic tests were analyzed on the basis of the patient's medical history.

## Results

16-year-old male patient 8 months prior to admission presented fatigue, weakness, weight loss and facial erythema with edema. On admission, the patient was confused, he had trembling extremities, scanning speech and a fever of 38,8 °C. Physical exam revealed muscle atrophy, lymphadenopathy, oral ulcers, echymosses, palmar erythema and malar rush. An infectious process was ruled out with viral panel and bacterial cultures. The blood count showed pancytopenia, increased ESR, AST and ASP, hypocomplementemia, hypertriglyceridemia and hyperferritinemia. Subsequently the patient presented antinuclear antibody in titer 1:20480 with a speckled pattern, positive anti-Sm and positive anti-ribosomal P protein antibodies. Bone marrow biopsy showed no changes typical for MAS. With this data the diagnosis of SLE was reached and treatment with pulses of glukocorticoids and an IVIG was started. During the next three days of hospitalization laboratory results were constantly getting worse despite the treatment and new clinical symptoms occurred (neurologic symptoms and bleeding from mucous membranes). Based on clinical manifestation and results of laboratory tests the patient was diagnosed with Macrophage Activation Syndrome secondary to SLE and the treatment with cyclosporine was added. Despite the clinical and laboratory tests improvement in the next five days the patient had a seizure attack followed by a cardiac arrest and after successful reanimation he was transferred to a intensive care unit. After two weeks the patient was again admitted to the clinic, he was conscious and respiratory stable although there was no logical contact, no reflexes in upper limbs, weak reflexes in lower limbs, profound muscle atrophy and hydrocephalus. Blood cell counts, markers of inflammation and other abnormalities in biochemical returned to normal.

## Conclusion

The diagnosis of MAS secondary to SLE is difficult due to common characteristics such as fever, pancytopenia, lymphadenopathy, neurological symptoms and skin manifestations. Careful assessment of laboratory results especially pancytopenia, hyperferritinemia and hypofibrinogenemia is crucial and leads to a quick and accurate diagnosis which is very important as MAS is considered to be a severe complication which puts patient's life at risk.

## Disclosure of interest

None declared.

